# Malarial Hemozoin Activates the NLRP3 Inflammasome through Lyn and Syk Kinases

**DOI:** 10.1371/journal.ppat.1000559

**Published:** 2009-08-21

**Authors:** Marina Tiemi Shio, Stephanie C. Eisenbarth, Myriam Savaria, Adrien F. Vinet, Marie-Josée Bellemare, Kenneth W. Harder, Fayyaz S. Sutterwala, D. Scott Bohle, Albert Descoteaux, Richard A. Flavell, Martin Olivier

**Affiliations:** 1 Department of Medicine, Microbiology and Immunology, Centre for the Study of Host Resistance, The Research Institute of the McGill University Health Centre, Montréal, Quebec, Canada; 2 Department of Immunobiology, Yale University School of Medicine, New Haven, Connecticut, United States of America; 3 Department of Laboratory Medicine, Yale University School of Medicine, New Haven, Connecticut, United States of America; 4 Institut National de la Recherche Scientifique-Institut Armand-Frappier, Laval, Quebec, Canada; 5 Department of Chemistry, McGill University, Montréal, Quebec, Canada; 6 Department of Microbiology and Immunology, University of British Columbia, Vancouver, Canada; 7 Inflammation Program, Department of Medicine, University of Iowa, Iowa City, Iowa, United States of America; 8 Howard Hughes Medical Institute, Yale University School of Medicine, New Haven, Connecticut, United States of America; Case Western Reserve University, United States of America

## Abstract

The intraerythrocytic parasite *Plasmodium—*the causative agent of malaria—produces an inorganic crystal called hemozoin (Hz) during the heme detoxification process, which is released into the circulation during erythrocyte lysis. Hz is rapidly ingested by phagocytes and induces the production of several pro-inflammatory mediators such as interleukin-1β (IL-1β). However, the mechanism regulating Hz recognition and IL-1β maturation has not been identified. Here, we show that Hz induces IL-1β production. Using knockout mice, we showed that Hz-induced IL-1β and inflammation are dependent on NOD-like receptor containing pyrin domain 3 (NLRP3), ASC and caspase-1, but not NLRC4 (NLR containing CARD domain). Furthermore, the absence of NLRP3 or IL-1β augmented survival to malaria caused by *P. chabaudi adami* DS. Although much has been discovered regarding the NLRP3 inflammasome induction, the mechanism whereby this intracellular multimolecular complex is activated remains unclear. We further demonstrate, using pharmacological and genetic intervention, that the tyrosine kinases Syk and Lyn play a critical role in activation of this inflammasome. These findings not only identify one way by which the immune system is alerted to malarial infection but also are one of the first to suggest a role for tyrosine kinase signaling pathways in regulation of the NLRP3 inflammasome.

## Author Summary

Malaria is widespread in the tropical and sub-tropical regions of the world, and is responsible for 2–3 million deaths annually. This disease is caused by parasites of the *Plasmodium* genus. The parasite feeds on the hemoglobin of red blood cells and generates a metabolic waste called hemozoin (Hz). Hz is released into the blood circulation during the rupture of red blood cells, which coincides with the production of many cytokines such as interleukin-1β (IL-1β), responsible in part for the periodic fever that is characteristic of the malaria disease. Here, we investigated how Hz activates macrophages (cells that engulf foreign material) to produce IL-1β. We found that Hz is taken up by macrophages initiating signals such as the tyrosine kinases Syk and Lyn that communicate to intracellular receptors. We also showed that Hz-induced IL-1β production is dependent on activation of the intracellular receptor NLRP3, the adaptor protein ASC and a protease called caspase-1 that cleaves IL-1β, therefore allowing it to be released from the cells. These findings not only identify one way in which the immune system is alerted to malarial infection but also dissect some of the signaling events triggered by Hz in the NLRP3 inflammasome pathway.

## Introduction

Malaria is a widespread infectious disease that affect up to 300 million individuals in the tropical and sub-tropical regions of the world, and is responsible for 2–3 million deaths annually [1]. Malaria is caused by parasites of the *Plasmodium* genus and is characterized by episodic fevers, anemia, headache and organ failure. *Plasmodium* parasites feed on erythrocyte hemoglobin and uses a heme detoxification mechanism that results in the formation of an insoluble, inert, dark-brown crystalline metabolic waste called hemozoin (Hz) [1],[2]. Hz is involved in the fever observed during the malaria process as intravenous injection of Hz caused thermal deregulation and was associated with the induction of pyrogenic cytokines [3]. In addition, the release of both *Plasmodium*-derived Hz and merozoites during the erythrocyte burst phase of the disease coincides with the massive induction of pro-inflammatory cytokines, such as IL-1β and TNF, and with the periodic fevers characteristic of malaria [3],[4].

IL-1β secretion is controlled by the recently described inflammasome, a signaling platform scaffold composed of NLR family members such as NLRC4 (NOD-like receptor containing CARD domain or IPAF) and members of the NLRP (NOD-like receptor containing pyrin domain) family including NLRP1 and NLRP3 (also known as NALP3 and cryopyrin). In addition, the NLRP3 inflammasome is composed of the adaptor molecule ASC (Apoptosis-Associated Speck-Like Protein) and the effector molecule caspase-1, the latter which is responsible for the cleavage of pro-IL-1β into its active form [5],[6]. TNF is induced by a wide variety of innate receptors but in particular by many members of the Toll-like receptors (TLR). It was previously reported that Hz can induce IL-1β secretion *in vitro* and *in vivo* [7],[8], however, TLRs are not required for the Hz-induced inflammatory response [9]. Given the clear association of IL-1β with the induction of fever and recent studies demonstrating that the NLRP3 inflammasome senses inorganic materials, such as monosodium urate (MSU, a gout-associated uric-acid crystals), silica, asbestos and aluminum hydroxide by producing IL-1β [6], we tested whether Hz can activate the NLRP3 inflammasome.

In addition, while NLRP3 ligands have been well identified, little is known about the upstream mechanisms that regulate its activation. Some mechanisms that have been proposed include efflux of potassium, increased intracellular calcium, reactive oxygen species (ROS) generation and lysosome disruption [6],[10]. However, having previously reported that both MSU and Hz can trigger production of inflammatory mediators via the activation of signaling cascades involving MAP kinase family members and various transcription factors, we have herein addressed the role of upstream signaling in the activation of the inflammasome that results in IL-1β production in response to the malarial pigment Hz.

## Results

### Hz-induced IL-1β and neutrophil recruitment is mediated by NLRP3, ASC and caspase-1, but not NLRC4

In these studies we utilized a chemically synthesized Hz to prevent contamination that could result from native Hz purification; the synthetic Hz is morphologically and chemically similar to native *Plasmodium*-isolated Hz (Fig. S1). Previously, we reported that both synthetic and native Hz induce similar expression profiles of chemokines and pro-inflammatory cytokines [7]. In addition, the synthetic Hz was subjected to elemental analysis to assess its purity. Theoretical calculated values of the molecular formula of Hz (C_68_H_62_N_8_O_8_Fe_2_) give 66.35% of carbon (C), 5.08% of hydrogen (H) and 9.10% of nitrogen (N). We have obtained elemental values from our synthetic Hz preparation very close with the theoretical one (C: 66.5%; H: 5.3%; N: 8.9%). To further show the purity of Hz, we performed an agarose gel with 200 µg of Hz and we did not detect any trace DNA or RNA contamination (Fig. S2A) and treatment with DNase or RNase did not interfere with Hz-induced IL-1β production (Fig. S2B). These data indicate that our synthetic Hz preparation is high purity and free of contaminant.

**Figure 1:**
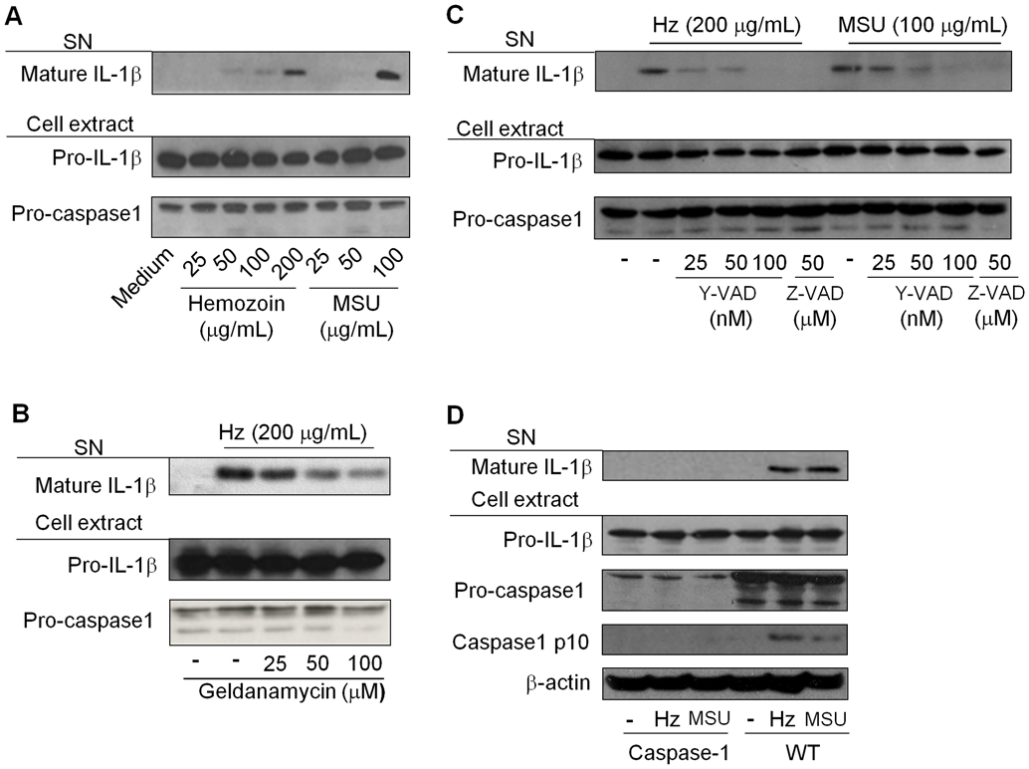
Hemozoin induces IL-1β maturation and secretion and is dependent on HSP-90 stability and caspase-1 activation. (A) PMA-differentiated THP-1 cells (0.75×10^6^ cells/0.5 mL) were stimulated with the indicated concentration of hemozoin (Hz) or Monosodium Urate (MSU) and (B) pre-treated or not with the HSP-90 inhibitor geldanamycin D or (C) the caspase-1 specific inhibitor Y-VAD-FMK or the broad caspase inhibitor Z-VAD-CHO. (D) Bone marrow derived macrophages – BMDM - (1.5×10^6^/mL) from either caspase-1-deficient or wild type (WT) mice were pre-treated with LPS (100 ng/mL) for three hours, washed and incubated with Hz (200 µg/mL) or MSU (100 µg/mL). After six hours of incubation, supernatant (SN) and cell extracts were collected and subjected to Western blot analysis with the indicated antibodies. Data show one experiment representative of three to five independent experiments.

To evaluate whether Hz activates the inflammasome, we measured IL-1β secretion by PMA-differentiated human monocytic cell line (THP-1) stimulated with increasing concentrations of Hz or MSU. Hz- and MSU-induced IL-1β production was found to be comparable (Fig. 1A). In accordance with previous studies showing that HSP-90 stability [11] modulates inflammasome assembly, we found that Hz-induced IL-1β secretion was reduced in the presence of the HSP-90 inhibitor geldanamycin D (Fig. 1B). Inhibition of caspase-1 activity using a specific competitor (Y-VAD-FMK) [12] or a broad caspase inhibitor (Z-VAD-CHO) also blocked Hz-induced IL-1β (Fig. 1C). To confirm the activation of caspase-1 we used the bone-marrow-derived macrophages (BMDM), since detection of the active form of caspase-1 in THP-1 cells is difficult as reported by others [13],[14]. Here, we show that Hz induced cleavage of caspase-1 to its enzymatically active (p10 subunit) form. BMDM were pre-stimulated with LPS in order to prime the induction of pro-IL-1β. As shown in Figure 1D, Hz and MSU, but not the pre-treatment with LPS, induced cleavage of caspase-1 and mature IL-1β production, which was completely abolished in BMDM from caspase-1 deficient mice.

**Figure 2:**
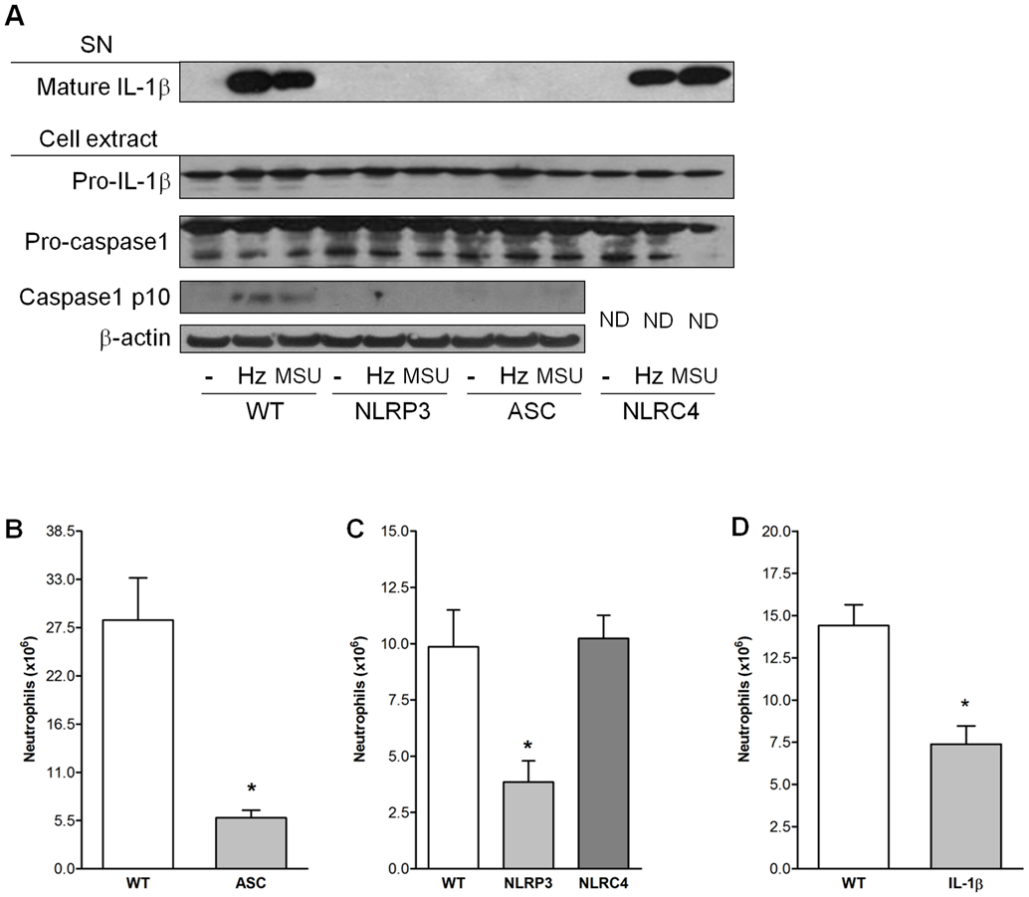
IL-1β production and neutrophil recruitment induced by hemozoin requires NLRP3, ASC and IL-1β but not NLRC4. (A) BMDM (1.5×10^6^ cells/mL) from wild type (WT), NLRP3-, ASC- or NLRC4-deficient mice were pretreated for three hours with LPS (100 ng/mL) for three hours, washed and stimulated with Hz (200 µg/mL) or MSU (100 µg/mL) where indicated. After six hours, supernatant (SN) and cell extracts were collected and subjected to Western blot analysis with the indicated antibodies. ND: not determined. (B) WT and ASC-deficient or (C) WT, NLRP3- and NLRC4-deficient or (D) WT and IL-1β-deficient mice were injected with 800 µg of hemozoin intraperitoneally in 1 mL PBS. After six hours, peritoneal cells were harvested, neutrophils were counted per total cell numbers and basal neutrophil influx (in PBS injected mice) was subtracted to determine total neutrophilic peritoneal recruitment. Data show one experiment representative of three independent experiments. Bars show mean+/−S.E.M., n = 4–6 mice/group. Unpaired Student's t-test was used to calculate P values (*p<0.05).

These results suggest a role for the inflammasome in Hz-induced IL-1β production. To further establish which intracellular receptors and/or adaptor proteins are activated by Hz, we used BMDM from mice deficient in NLRP3, ASC or another NLR, NLRC4 (NLR containing CARD domain, also known as IPAF). We found that Hz- and MSU-induced caspase-1 activation and IL-1β maturation were dependent on NLRP3 and ASC but not NLRC4 (Fig. 2A). On the other hand, macrophages from NLRC4 mice failed to respond to *Salmonella typhimurium* infection (Fig. S3). To evaluate whether activation of the NLRP3 inflammasome is involved in Hz-induced inflammatory responses *in vivo*, mice were injected intraperitoneally with Hz and then neutrophil recruitment to the site of injection was examined. Hz induced significant recruitment of neutrophils to the peritoneal cavity in wild type, but not in ASC-deficient (Fig. 2B) or in NLRP3-deficient mice (Fig. 2C). As expected, NLRC4 was not involved in the inflammatory response induced by Hz (Fig. 2C). We further investigated whether IL-1β directly contributed to the recruitment of neutrophils. As expected, IL-1β deficient mice showed a significant decrease in the number of neutrophils elicited by Hz stimulation (Fig. 2D). However, we did not observe a complete abrogation of neutrophil influx as previously seen with IL-1 receptor-deficient mice stimulated with other inflammasome ligands [15]. These results suggest that a portion of the Hz-induced inflammatory response *in* vivo may results from other ligands of the IL-1 receptors and/or other cytokines and chemokines known to be induced by Hz [3],[7],[8].

### NLRP3 and IL-1β-deficient mice show increased survival and lower body temperature when infected with the malarial parasite *Plasmodium chabaudi adami* DS

**Figure 3:**
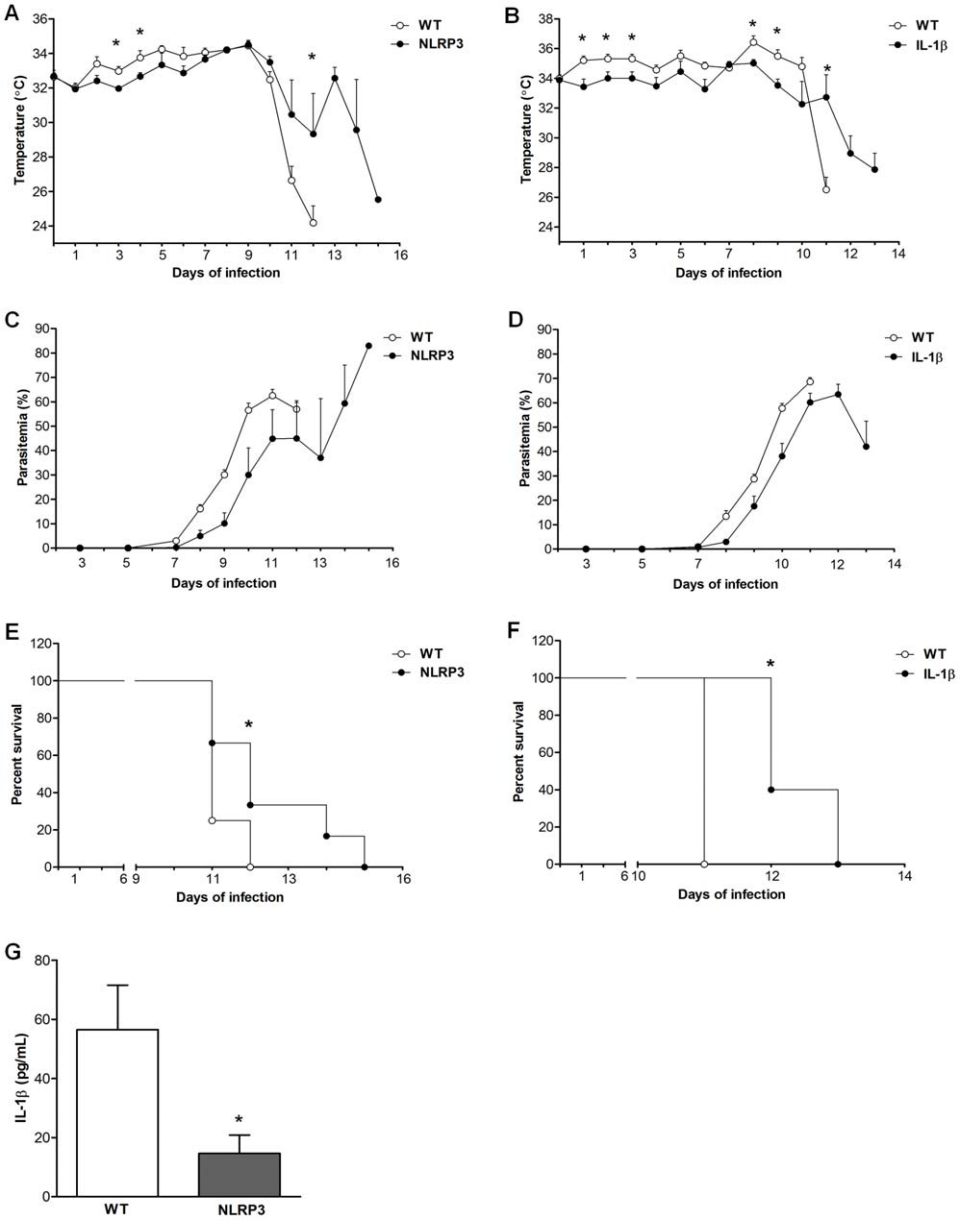
NLPR3- and IL-1β-deficient mice show increased survival to malaria infection and reduced fever. Wild type (WT), NLRP3- or IL-1β-deficient mice were infected with *Plasmodium chabaudi adami* strain DS and after the indicated time, the mouse body temperature (A and B), parasitemia (C and D), and survival of mice (E and F) were monitored. IL-1β was measured in serum collected before death (G), dashed line represents ELISA detection limit. Statistical differences (A, B and G) were estimated using *t* test. Statistical significances between survival curves were determined using the Mantel–Haenszel test. *p<0.05. Results from a representative infection experiment (n = 5–8) are shown.

Thus far, we have shown that Hz-induced IL-1β production is dependent on the NLRP3 inflammasome, in addition, it is known that IL-1β is involved in malarial fever [4]. To evaluate the role of IL-1β and the NLRP3 inflammasome during malarial disease we infected IL-1β- and NLRP3-deficient mice with *Plasmodium chabaudi adami* DS, which is a mouse virulent strain. Of interest, both IL-1β- and NLRP3 mice presented a slight but significant lower body temperature (Fig. 3A and 3B) and parasitemia (Fig. 3C and 3D) in the early phase of infection. These knockout mice also showed a significantly prolonged survival compared with wild type mice, but ultimately succumbed to the infection (Fig. 3E and 3F). Finally, in the late phase of infection, the level of IL-1β was significantly lower in NLRP3-deficient mouse in comparison with wild type mice (Fig. 3G) and was not detectable in IL-1β-deficient mouse (data not shown). These results indicate that IL-1β is an important factor in the pathophysiology during malaria infection.

### Hz-induced IL-1β requires phagocytosis, ROS generation, potassium efflux and cathepsin B activation without phagosome damage

**Figure 4:**
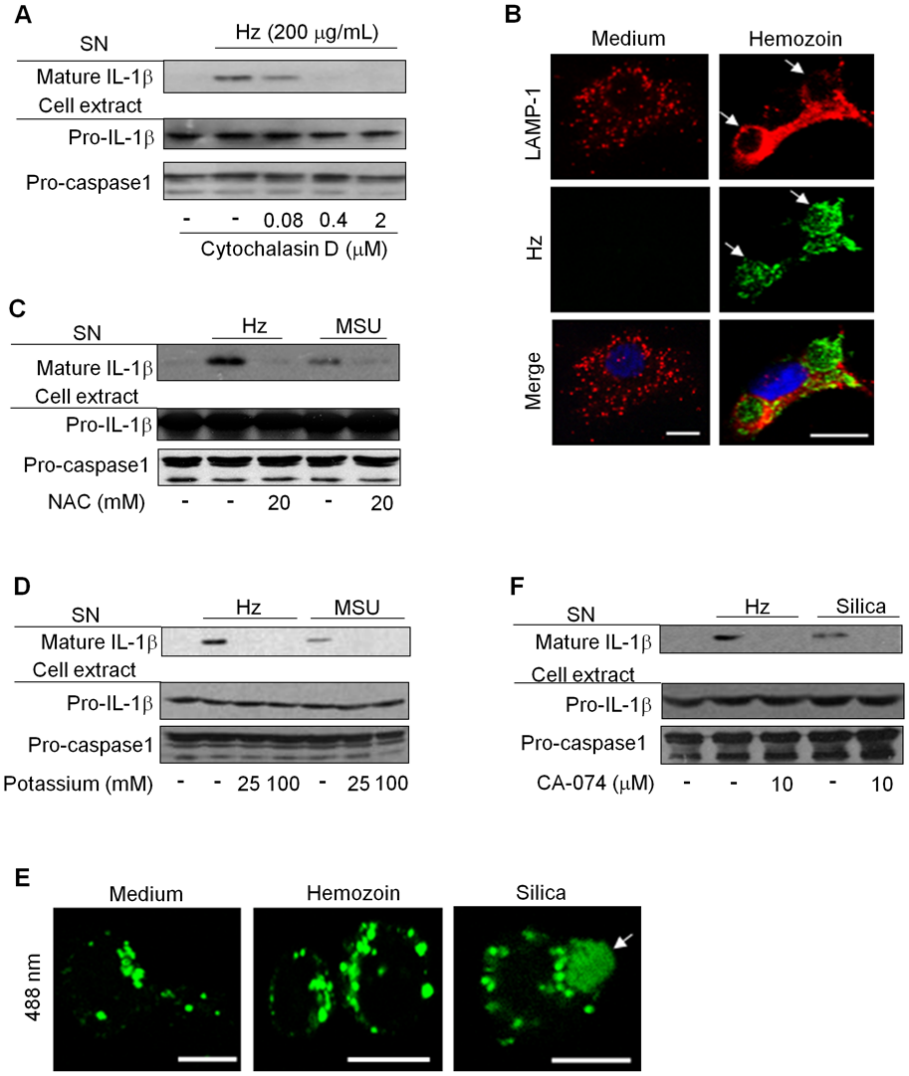
Hemozoin-induced IL-1β production is dependent on phagocytosis, reactive oxygen species (ROS) generation, potassium efflux and cathepsin B. PMA-differentiated THP-1 cells (0.75×10^6^ cells/0.5 mL) were stimulated with Hz (200 µg/mL) or MSU (100 µg/mL) and exposed to the indicated concentrations of (A) phagocytosis inhibitor cytochalasin D, (C) the ROS-scavenger N-acetyl cysteine (NAC), (D) extracellular potassium or, (F) the cathepsin B inhibitor CA-074. After six hours of incubation, supernatant (SN) and cell extracts were collected and subjected to Western blot analysis with the indicated antibodies. (B) BMDM were incubated or not with 200 µg/mL Hz (green) and stained for LAMP-1 (red) and for nucleus with DRAQ5 (blue). (E) PMA-differentiated THP-1 cells were stimulated or not with Hz (200 µg/mL) or silica (400 µg/mL) in the presence of DQ-OVA (10 µg/mL) for 30 minutes, washed and further incubated for three more hours. Green fluorescence represents cleaved OVA. Data shown are images obtained by confocal microscopy from one representative experiment of three independent experiments. Scale bars = 5 µm.

Hz is rapidly engulfed by phagocytes, both in infectious and experimental conditions [2]. Therefore, to test the importance of phagocytosis on Hz-induced IL-1β production, cells were treated with cytochalasin D - a powerful actin polymerization inhibitor - prior to the addition of the crystals. Consistent with other crystals that induce inflammasome activation [15],[16],[17], we found that Hz-induced IL-1β seems to be dependent on its internalization (Fig. 4A). Furthermore, under certain conditions phagocytosis requires cholesterol-rich lipid domains [18] and as expected, cholesterol depletion by MβCD inhibited HZ-induced IL-1β (Fig. S5A), which was due to the disruption of lipid rafts (Fig. S5C). Further characterization of Hz phagocytosis by confocal immunofluorescence microscopy revealed that Hz was internalized in a vacuole that acquired lysosomal features, as shown by the presence of Lamp-1 surrounding the engulfed Hz phagosomes (Fig. 4B). Phagocytosis is generally accompanied by the generation of reactive oxygen species (ROS), which modulates inflammasome activation by crystals such as silica [19], MSU [15] and asbestos [20]. Since Hz induces ROS production [7] its requirement in Hz-induced IL-1β production was evaluated. The ROS scavenger, N-acetylcysteine (NAC) inhibited both Hz- and MSU-induced IL-1β production (Fig. 4C), which suggests a potential upstream role for ROS in inflammasome activation by Hz. Cellular potassium efflux is another critical step in inflammasome activation induced by all known NLRP3 activators [21],[22]. As shown in the Figure 4D, inhibition of potassium efflux by high concentrations of extracellular potassium decreased IL-1β production induced by Hz. The above results suggest that Hz shares a common mechanistic pathway in the activation of the NRLP3 inflammasome with classical triggers such as ATP and others insoluble crystals [21],[23].

Recently, lysosomal destabilization has been proposed as one mechanism whereby inorganic materials such as silica and aluminum hydroxide activate the inflammasome [17]. To assess lysosomal morphology in the context of Hz stimulation, we performed a confocal analysis of PMA-matured THP-1 cells loaded with a self-quenched conjugate of ovalbumin (DQ-OVA) that fluoresces only upon proteolytic degradation. We found that Hz did not affect the shape of lysosomes in comparison to untreated cells. In contrast, silica-treated cells contained swollen lysosomes (Fig. 4E), suggesting that Hz may activate the inflammasome through distinct, but related pathway. Indeed, inhibition of the lysosomal cysteine protease (cathepsin B) by the specific inhibitor CA-074 abrogated IL-1β induced by Hz and silica (Fig. 4F) [17]. However, it is still unclear how this enzyme is involved in inflammasome activation and indeed, many of the proximal signaling events in NLRP3 and NLR activation remain unknown.

### Hz-induced upstream signaling regulates IL-1β production

**Figure 5:**
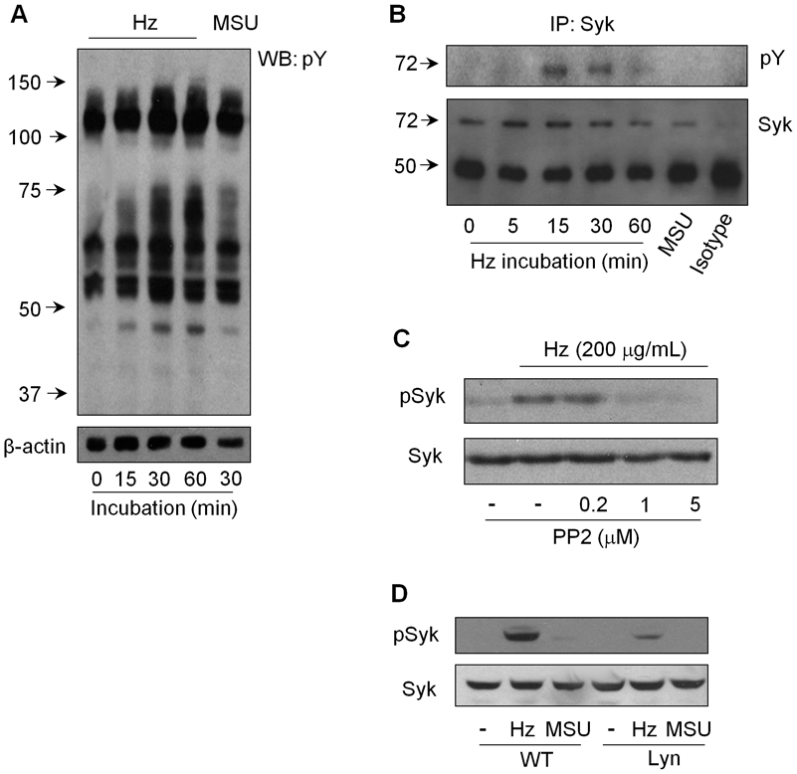
Hz induces Syk phosphorylation dependent on Src kinases. PMA-differentiated THP-1 cells (0.75×10^6^ cells/0.5 mL or 10×10^6^ cell per immunoprecipitation - IP) were stimulated with Hz (200 µg/mL) or MSU (100 µg/mL) for the indicated time or 30 min if not indicated and (A) cell lysates or (B) samples from IP with a specific antibody to Syk or a matched isotype control were subjected to western blot analysis to phosphorylated tyrosine residues (pY). (C) Cells were pre-treated with the Src inhibitor PP2. (D) BMDM (1.5×10^6^ cell/mL) from wild type (WT) or Lyn-deficient mice were pretreated for three hours with LPS (100 ng/mL), washed and treated or not with Hz (200 µg/mL) or MSU (100 µg/mL) for 30 minutes. IP samples or total cell lysates were subjected to Western blot analysis with the indicated antibodies. Numbers to the left of blots represent protein size in kDa.

Whereas we obtained clear evidence that Hz can induce IL-1β production in an inflammasome-dependent manner that required active cathepsin B, we did not find evidence of Hz-induced lysosomal rupture as previously reported with silica [17]. Release of cathepsin B without lysosomal rupture has been observed in monocytes treated with the potassium ionophore nigericin [24]. In addition, the widely expressed Spleen Tyrosine Kinase (Syk) was shown to be required for cathepsin B release into the cytosol in a model of B cell receptor-mediated apoptosis [25]. We therefore screened Hz-activated macrophages for changes in their tyrosine phosphorylation profiles. Consistent with the possible involvement of Syk, we observed a band with an apparent molecular weight of 72 kDa that was phosphorylated in response to Hz, but not MSU (Fig. 5A). We then carried out anti-Syk immunoprecipitation, followed by anti phospho-tyrosine analysis and found that Syk was phosphorylated in response to Hz, but not MSU stimulation (Fig. 5B). Even by extending the time-course of stimulation, MSU did not induce Syk phosphorylation (Fig S4A).

Syk is typically activated via receptors or adaptor proteins containing immunoreceptor tyrosine-based activation motifs (ITAMs) or ITAM-like domains phosphorylated by Scr family kinases following receptor clustering [26],[27]. The Src kinase inhibitor PP2 decreased the Hz-induced Syk phosphorylation in a dose dependent manner (Fig. 5C). Syk activation can be mediated by the Scr family kinase member Lyn [28]. Lyn is typically found in lipid raft signaling platforms and disruption of these rafts by MβCD (Fig. S5C) indeed blocked, in dose-dependent manner, Syk phosphorylation in Hz-stimulated monocytes (Fig. S5B). Using BMDM from Lyn-deficient mice, we found that Hz-induced Syk phosphorylation required Lyn, and further confirmed that MSU does not utilize this signaling pathway in either murine or human macrophages (Fig. 5).

**Figure 6:**
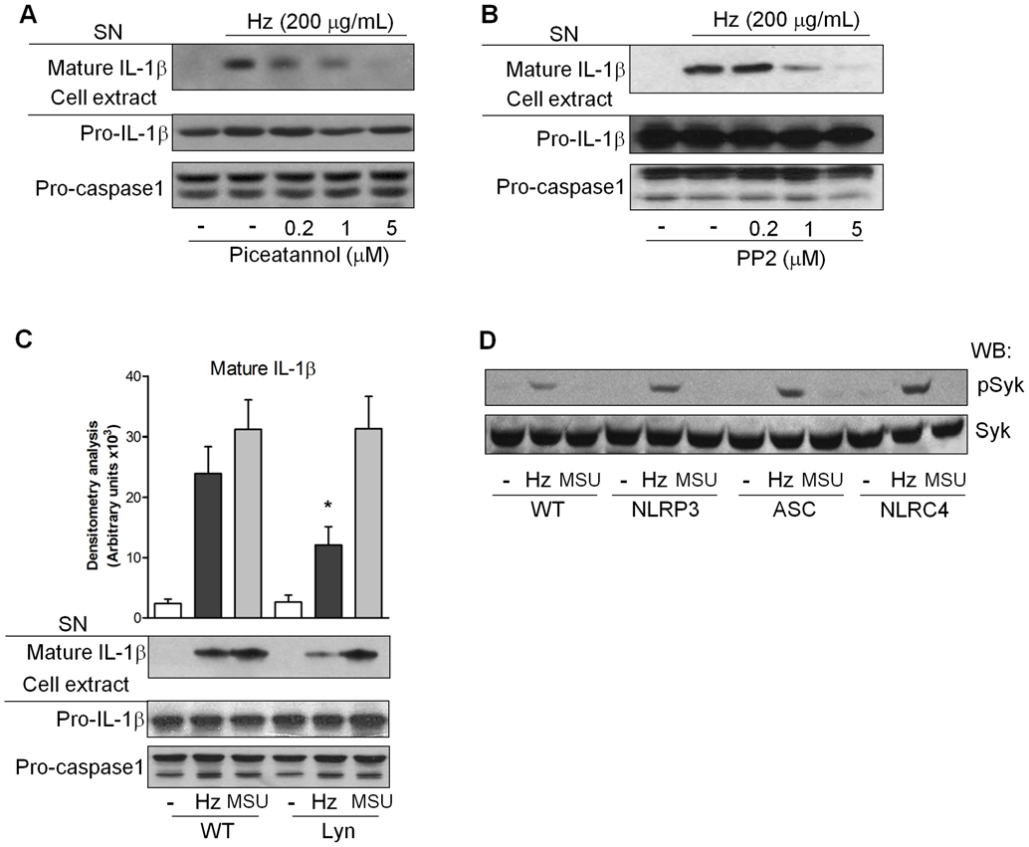
Syk and Src kinases regulate IL-1β production induced by hemozoin. PMA-differentiated THP-1 cells (0.75×10^6^ cells/0.5 mL) were pretreated with either the SYK inhibitor piceatannol (A) or the Src inhibitor PP2 (B). BMDM (1.5×10^6^ cell/mL) from wild type (WT) or Lyn-deficient mice were pretreated for three hours with LPS (100 ng/mL), washed and treated or not with Hz (200 µg/mL) or MSU (100 µg/mL) for six hours (C). Supernatant (SN) or total cell lysates were subjected to Western blot analysis with the indicated antibodies. Numbers to the left of blots represent protein size in kDa. (C) Bars show mean+/−S.E.M. of densitometry of three independent experiments. *p<0.05 comparing Lyn-deficient *vs.* WT mice. BMDM were treated as described in the legend of Fig. 2A were stimulated for 30 minutes and data show one experiment representative of three to five independent experiments (D).

Next we evaluated the role of Lyn and Syk in Hz-induced IL-1β production. IL-1β secretion stimulated by Hz was inhibited in macrophages treated with the Syk inhibitor piceatannol (Fig. 6A), the Scr kinase inhibitor PP2 (Fig. 6B), and more specifically using Lyn-deficient BMDM (Fig. 6C). Importantly, in this last experiment, Hz-induced IL-1β production was only partially inhibited, which suggest that another member of the Src kinase family could play the same role of Lyn, since these kinases are known to be functionally redundant [28]. Of note, MSU-induced IL-1β production was not affected in Lyn-deficient BMDM pre-treated with LPS. To evaluate the relative roles of LPS and Hz in the induction of this signaling pathway, we treated BMDM with LPS and we observed that LPS by itself did not induce phospho-Syk, and indeed pre-treatment with LPS reduced Hz-induced Syk phosphorylation (Fig. S4B). Furthermore, Hz-induced Syk activation is not affected by the absence of the MyD88 adaptor protein (Fig. S4C). However, MyD88-deficient cells show a delay in the phosphorylation of c-jun N-terminal kinase (JNK) stimulated by LPS (Fig. S4C), similar as previously reported [29]. These results rule out a possible effect of LPS on Syk phosphorylation. Consistent with the involvement of this kinase in a pathway upstream of the inflammasome, NLRP3-, ASC- and NLRC4-deficient macrophages exhibited normal Syk phosphorylation upon Hz stimulation (Fig. 6D).

**Figure 7:**
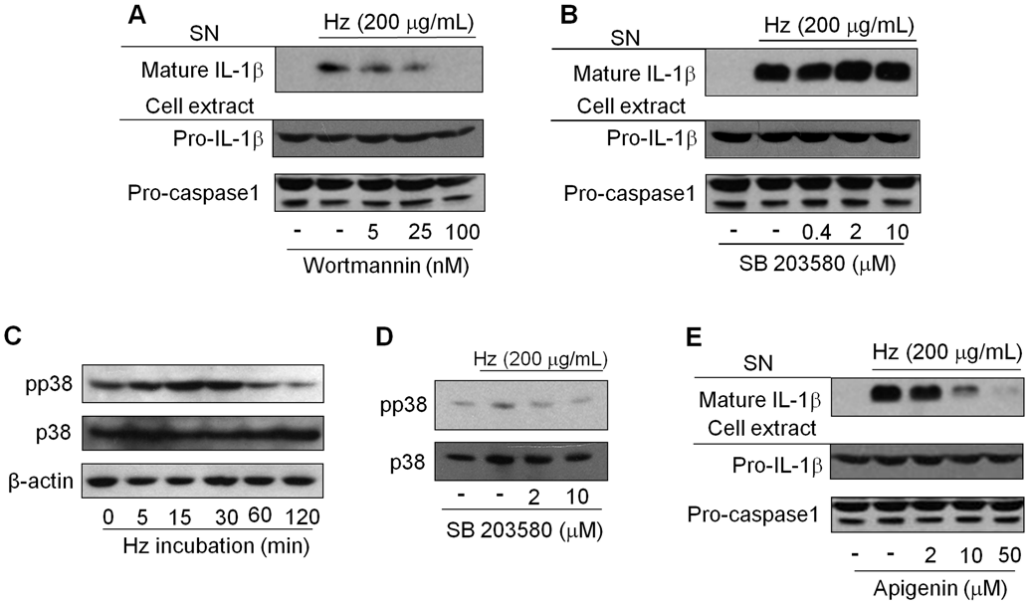
The role of other kinases in hemozoin-induced IL-1β. PMA-differentiated THP-1 cells (0.75×10^6^ cells/0.5 mL) were pretreated with: (A) PI3K inhibitor – wortmannin, (B and D) p38 inhibitor - SB 203580, or (E) ERK inhibitor - apigenin followed by Hz (200 µg/mL) stimulation for six hours (IL-1β) or 30 minutes (pp38) or the indicated time (C). Supernatant (SN) and cell lysates were subjected to Western blot analysis with the indicated antibodies. Data show one experiment representative of two to five independent experiments.

Syk activates various downstream signaling pathways, including phosphoinositide 3-kinase (PI3K) [30] and extracellular signal-regulated kinase (ERK). To test whether the PI3K pathway is required for propagation of the Syk signaling pathway following Hz exposure, the PI3K inhibitor wortmannin was used prior to Hz stimulation. Inhibition of PI3K indeed abrogated IL-1β maturation (Fig. 7A). We have previously identified MAPK activation upon Hz stimulation of macrophages [31]. We therefore attempted to isolate which pathways might be required for Hz-induced IL-1β production using known p38 and ERK kinase inhibitors. Whereas p38 phosphorylation can be observed following Hz stimulation, inhibition of p38 with SB203580 failed to block Hz-induced IL-1β production (Fig. 7B–D). On the other hand, inhibition of ERK with Apigenin abrogated Hz-induced IL-1β secretion (Fig. 7E). Altogether, these results reveal that Lyn/Syk activation following Hz exposure initiates the PI3K and ERK signaling pathways and these pathways appear to regulate the production of mature IL-1β.

**Figure 8:**
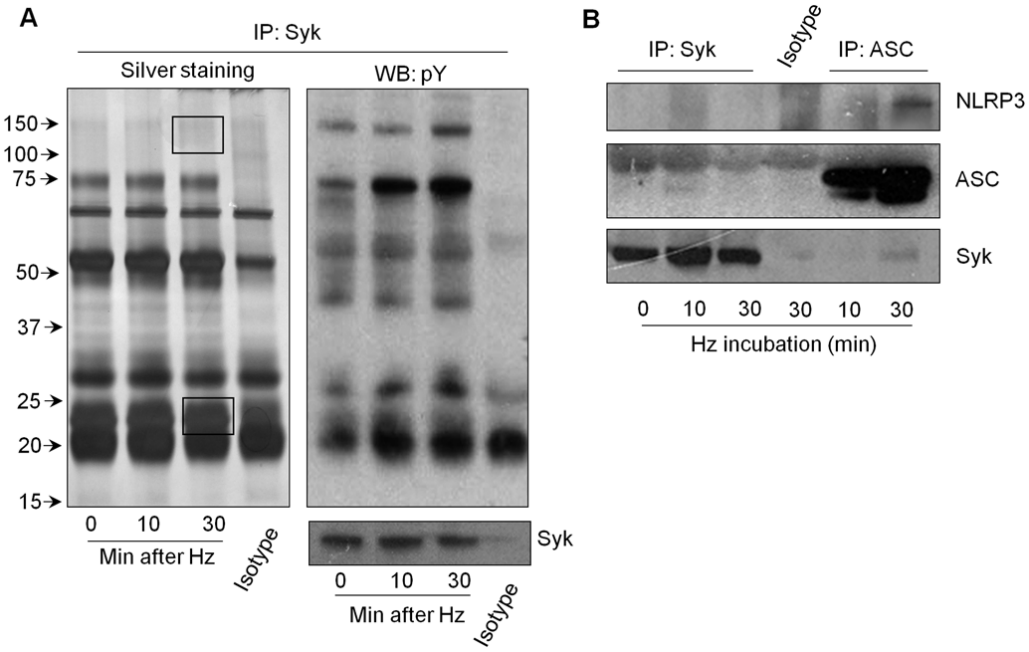
Syk complexes with inflammasome proteins. PMA-differentiated THP-1 cells (10×10^6^ cells per immunoprecipitation - IP) were stimulated with Hz (200 µg/mL) for the indicated time. Lysates were immunoprecipitated with a specific antibody to SYK or matched isotype control and samples were subjected to (A left panel) silver staining or (A right panel) to Western blot analysis to phosphorylated tyrosine residues (pY). Squares in A left panel represent the bands excised and analyzed with LC-MS/MS. (B) Samples from IP with a specific antibody to Syk, ASC or matched isotype control and samples were subjected to Western blot (WB) analysis with specific antibody for Syk, ASC or NLRP3. Numbers to the left of blots represent protein size in kDa. Data show one experiment representative of three independent experiments.

While a number of stimuli are known to activate the NLRP3 inflammasome, there is no evidence that NLRP3 directly recognizes these ligands. Therefore an indirect pathway of NLRP3 activation is likely, however the identity of the direct molecular switch of NLRP3 has not been identified. Our studies provide the first evidence for a role of tyrosine kinase signaling molecules in NLRP3 activation. To examine whether Syk can modulate the inflammasome by directly interacting with its components, we immunoprecipitated Syk and then immunoblotted for potential partners associated with Syk by silver staining and western blotting (Fig. 8A). Selected differential bands were analyzed by LC-tandem mass spectrometry. Interestingly, two to three different peptides covering 11–23% of the Pyrin domain (Pyd) [32] were identified. Pyrin domains are known to mediate protein-protein interactions and are crucial in many of the NLR inflammasome complexes, and in particular, mediate the NLRP3 and ASC interaction [6]. We therefore confirmed by western blotting whether NLRP3 or ASC can be co-immunoprecipitated (co-IP) with Syk. Whereas NLRP3 was shown to weakly interact with Syk, ASC was found to strongly associate with this kinase upon Hz stimulation (Fig. 8B). These findings suggest that Syk, and possibly other unidentified signaling kinases, can associated with the ASC/NRLP3 inflammasome.

**Figure 9:**
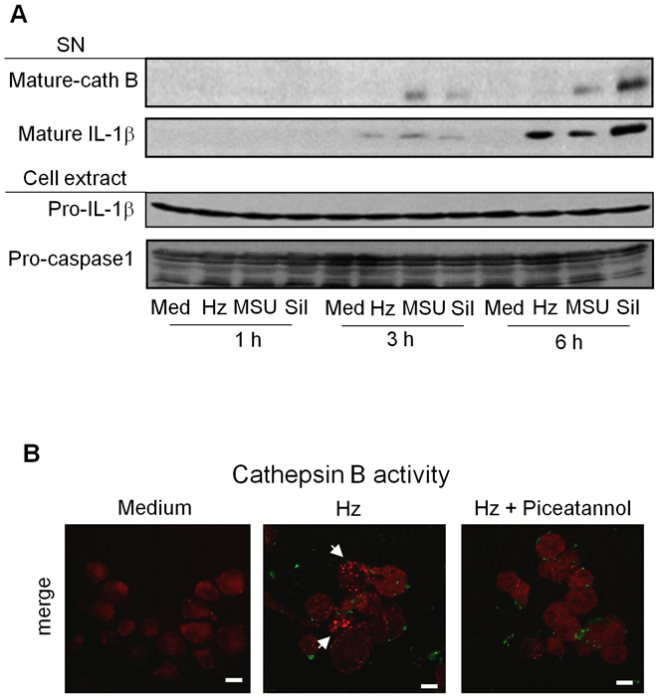
Hz-activated Cathepsin B is regulated by Syk. PMA-differentiated THP-1 cells (0.75×10^6^ cells/0.5 mL) were stimulated with Hz (200 µg/mL), MSU (100 µg/mL) or silica (Sil, 400 µg/mL). After different times of incubation, supernatant (SN) and cell extracts were collected and subjected to Western blot analysis with the indicated antibodies (A). PMA-differentiated THP-1 cells (0.2×10^6^ cells/0.5 mL) were pre-treated (30 min) with 5 µM of piceatannol and incubated or not with 200 µg/mL Hz (green) and cathepsin B activity was detected using a red fluorescence substrate of cathepsin B. Data shown are images obtained by confocal microscopy from one representative experiment of three independent experiments. Scale bars = 5 µm (B).

Another possible mechanism is that Syk could be controlling the NLRP3 inflammasome by regulating cathepsin B activation. First, we tested if Hz can induce release of the active form of cathepsin B in the supernatant and as showed in the Figure 9A, Hz did not induce cathepsin B release into supernatant as has been observed with MSU and silica. However, using a cathepsin B substrate that emits red fluorescence upon cleavage we demonstrated that Hz induces rapid (30 min) and transient (maximum 1.5 h) intra-compartmental cathepsin B activation that was dependent on Syk activation (Fig. 9B). These results indicate that Syk not only can associate with the inflammasome component but it can also modulate cathepsin B activation.

## Discussion

It has been described that NLRP3 senses many crystalline materials that are involved in inflammatory diseases, such as MSU [15], silica [19], and asbestos [20]. Here we provide the first demonstration that the malaria pigment hemozoin (Hz) can also activate the NLRP3 inflammasome. Importantly, the Hz concentration shown to activate the NLRP3 inflammasome *in vitro* is similar in range to the concentration of Hz in the blood of patients with moderate parasitemia [8],[33]. Moreover, it was never shown in the previous studies that direct contact between a crystal and NLRP3 is necessary to induce activation. Similarly, we found that Hz does not translocate from the phagosome/lysosome compartment to the cytoplasm, as it is located within LAMP-1-positive compartments, suggesting that Hz activated the NLRP3 inflammasome in an indirect manner.

It has been proposed that the NLRP3 inflammasome senses not only pathogen-associated molecular patterns but also danger signals such as stress-related molecules [5]. In agreement, here we show that Hz-induced IL-1β production was dependent on ROS generation and potassium efflux into the cytoplasm. In addition to previous studies on the inflammasome, we further identified an upstream signaling pathway involving the Src kinase Lyn, the tyrosine kinase Syk and Syk-downstream kinases such as PI3K and ERK that collectively appear to be involved in the regulation of Hz-induced IL-1β production. Simultaneously to us, it has been recently reported that Syk kinase is involved in upstream signaling of NLR inflammasome triggered by fungi [34]. Whether these findings represent a general regulatory mechanism of this intracellular innate immune response will need further investigation.

The Lyn/Syk pathway appears to be uniquely activated in the innate response to Hz crystals, as opposed to other NLRP3-activating crystals such as MSU. In our hands, MSU did not induce Syk or Lyn phosphorylation in PMA-differentiated THP-1 cells nor in BMDM. However, MSU was previously reported to trigger Syk phosphorylation in dendritic cells [35] and human neutrophils [36], as well as Lyn phosphorylation in neutrophils [37].

An intriguing question is how this signaling cascade may modulate the inflammasome/IL-1β production. For instance, we found some indication that Syk can interact with ASC, but not NLRP3. ASC, as it is well known, interacts with NLRP3. These results suggest that Syk may modify ASC. In support of this finding, there is evidence that the ASC pyrin domain can be phosphorylated [38]. Moreover, hyperphosphorylated PSTPIP1 (proline serine threonine phosphatase-interacting protein) was shown to interact with the pyrin protein [39], resulting in its conformational change and further its interaction with ASC [40]. Another possible mechanism whereby kinases can modulate IL-1β production is by modulating intracellular calcium concentration or cathepsin B activation. Syk is involved in the activation of intracellular calcium mobilization in other models [41]. In fact, increased calcium concentrations have been found to modulate inflammasome activation by different stimuli such as MSU and UV radiation [22],[42]. Finally, Syk was found to control the activation of cathepsin B and Hz-induced IL-1β production was dependent on cathepsin B activation, similar to other inflammasome activators such as silica, MSU [17] or nigericin [24]. We showed that specific inhibition of Syk blocked the Hz-induced cathepsin B activation. Collectively, it is clear that different steps in the Hz-induced IL-1β production can be regulated by intracellular signaling. However, further study will be necessary to better characterize these regulatory events in regards to the different inorganic crystals that can trigger NLRP3 inflammasome activation.

Another interesting observation is that Hz-activated cathepsin B occurred in the intracellular compartment and is rapidly quenched (1–3 hours), suggesting either a transient activation or cathepsin B release into the cytosol. The idea of transient activation of cathepsin B by Hz is supported by the absence of cathepsin B in the supernatant of cells stimulated with Hz and the absence of lysosomal damage upon Hz treatment. The mechanism utilized by Hz-activated cathepsin B to modulate the inflammasome remains unclear. However, a possible mechanism is that cathepsin B can activate directly caspase-1 as it has been shown in previous works [17],[24]. Of interest, both caspase-1 and cathepsin B, in addition to inflammasome components and IL-1β are found in multivesicular bodies surrounded by LAMP-1 [43]. It is known that Syk and Syk-activated downstream kinases such as PI3K regulate the trafficking of intracellular vesicles [44]. In this way, Hz-induced Syk might be controlling not only the inflammasome cascade but also the trafficking of multivesicles.

The Lyn/Syk activation finding raises the intriguing possibility that an as yet unidentified receptor or adaptor protein containing an ITAM or ITAM-like domain, such as Dectin-1, TREM family members, Siglec or DAP12 [26],[27], might be activated upon Hz stimulation to trigger the signaling cascade involved in inflammasome activation. However, a recent work with dendritic cells demonstrated that MSU did not require a surface receptor - instead the crystals interact with surface lipid rafts and this was enough to trigger Syk/PI3K pathway [35]. In our study, we have demonstrated that lipid rafts are involved in the Hz-induced signaling pathway and IL-1β production. Other potential receptors that could mediate Hz-triggered signaling are the Toll-like receptors (TLR). However, we have recently demonstrated in collaboration with Parroche and colleagues [9] that Hz alone fails to activate TLRs except when Hz is coated with parasitic DNA and consequently activating TLR9. Similarly, we also observed that HEK293 cells transfected with different TLRs were not activated by Hz although these cells were able to induce NF-κB activation following specific ligand stimulations (Jaramillo and Olivier, unpublished data). We also showed that the MyD88 signaling pathway is not involved in the Hz-induced Syk phosphorylation. Experiments to identify surface receptors or lipids that recognize Hz are currently underway.

In the present work we further supported the role of NLRP3-mediated IL-1β production in Hz-mediated inflammatory cell recruitment using IL-1β deficient mice. Apart from its inflammatory role, IL-1β is a pyrogenic cytokine that in small concentrations induces the production of other cytokines such as IL-6 and can cause hypertension and fever [45]. In fact, we showed that NLRP3- and IL-1β-deficient mice exhibited lower body temperature during the early phase of *P. chabaudi Adami* infection. Hz-induced IL-1β can be the mediator of the up-regulation of chemokines and cytokines during malaria infection, which is independent of TLRs but dependent on MyD88 [46]. This suggests that another MyD88 dependent receptor such as IL-1R is involved and supports a role for IL-1β in malaria-related pathology. Corroborating this hypothesis, we showed that IL-1β- and NLRP3- deficient mice showed a better survival than wild type mice in murine experimental model of malaria. Not surprisingly, it was not sufficient to provide full protection likely due to the complexity of malarial disease, which is under the regulation of many different receptors, cytokines, signaling events and physiological features.

Collectively, our study provides the first demonstration that a malarial-derived metabolic product, namely hemozoin, can induce NLRP3 inflammasome activation and IL-1β production though the involvement of the Src kinase Lyn and the tyrosine kinase Syk. However, excessive IL-1β secretion can be deleterious to the host; in fact, we observed that higher production of IL-1β correlates with early death in murine experimental malaria. Therefore these findings strongly support the fact that Hz is critical in malaria pathology. A better understanding of the molecular and cellular events regulating malaria inflammatory-related pathologies may provide new insights into the design of treatments aimed at reducing the exaggerated inflammatory disorders and debilitating sequelae.

## Materials and Methods

### Animals

With the subheading Ethics Statement, all protocols used in this study were approved by the Institutional Animal Care and Use Committees at the McGill University or Yale University. IL-1β- and Lyn-deficient mice were provided by Dr. G.Sébire and Dr. K. W. Harder (University of Sherbrooke, Quebec and University of British Columbia, Vancouver, Canada), respectively. The generation of IL-1β-, Lyn-, NLRP3-, ASC-, caspase-1-, and NLRC4-deficient mice has been described previously [47],[48],[49],[50],[51]. Caspase-1-, ASC-, and NLRP3-deficient mice were backcrossed onto the C57BL/6 genetic background for at least nine generations. NLRC4-deficient mice were backcrossed onto the C57BL/6 genetic background for at least six generations. Age- and sex-matched C57BL/6 mice purchased from the National Cancer Institute or Charles River were used as WT controls.

### Reagents and cells

Hemin (>99% of purity) was purchased from Fluka; RPMI-1640 medium, Penicillin-Streptomycin-Glutamine (PSG) from Wisent, fetal bovine serum (FBS), Alpha MEM medium from Gibco; CV-Cathepsin B detection kit, PP2, piceatannol, geldanamycin, cytochalasin D, Y-VAD-FMK and Z-VAD-CHO from Biomol; MSU, anti-human NLRP3 and ASC from Alexis Biochemical; inhibitor protease cocktail from Roche; CHAPs from Fisher; A/G-coupled agarose beads, anti-human pro-IL-1β, anti-human or murine caspase-1 and anti-Syk from Santa Cruz; True Blot anti-rabbit Ig, anti-phosphoY/HRP from eBioscience; PVDF from Bio-rad; anti-LAMP-1 Ab from Developmental Studies Hybridoma Bank at the University of Iowa; anti-human mature IL-1β, anti-pp38 and anti-p38 from Cell signal; anti-pSyk and anti-pY (4G10) from Upstate; rat or goat anti-murine IL-1β and recombinant IL-1β from R&D system; DQ-OVA from Invitrogen; anti-rat AlexaFluor 568, cholera toxin B-AlexaFluor 568 from Molecular Probes; DRAQ5 from Biostatus; Fluoromount-G from Southern Biotechnology; all others unlisted or not indicated reagents were purchased from Sigma. L929 and THP-1 cell line from ATCC. MyD88 KO BMDM was generated from MyD88-deficient mice and kindly supplied by Dr. Danuta Radzioch (McGill University, Montreal, Canada).

### Native and synthetic hemozoin production

Native and Synthetic Hz have been obtained as previously described [8],[31]. We have modified synthetic Hz preparation, using high purity chemical reagents (>99% of purity), as follows: 0.8 mmol Hemin was dissolved in degassed NaOH (0.1 M) for 30 minutes with mild stirring. pH 4.0 was adjusted adding drop-wise propionic acid. The mixture was allowed to anneal at 70°C for 18 hours. Then washed three times with NaHCO_3_ (0.1 M) for three hours and the last wash with MeOH. All washes were alternated with distilled H_2_O. Finally, the sample was then dried in a vacuum oven overnight over phosphorous pentoxyde. All synthetic hemozoin samples were analyzed by X-ray powder diffraction, field emission gun scanning electron microscopy, and infra-red spectroscopy to characterize the crystalline state of Hz. Hz purity was assessed by elemental analysis [52].

### THP-1 culture and stimulation

THP-1 cells (ATCC) were cultured with RPMI-1640 medium supplemented with 10% FBS, 1% PSG, 50 µM of 2-β-mercaptoetanol, Glucose 4.5 g/L and 1 mM sodium pyruvate. THP-1 differentiation: (1.5×10^6^ cells/mL) were incubated with 0.5 µM of PMA, after three hours cells were washed and plated at 0.75×10^6^ cells/mL or 0.2×10^6^ cell/0.5 mL in 12 well plates (IL-1β) or 24 well plates containing coverslips (confocal) and incubated for 20–24 hours. This treatment increases the phagocytic properties of the cells and induces a constitutive production of pro-IL-1β. Prior to stimulation, cells were washed and 500 µL of Alpha MEM medium without FBS was replaced. Cells were pre-treated with different drugs for 1 hour and stimulate with Hz, MSU or silica as indicated in figure legends.

### Mouse infection

Gender and age matched wild type (WT), NLRP3- or IL-1β-deficient mice were injected i.p. with 5×10^4^ *Plasmodium chabaudi adami* DS infected red blood cells obtained from syngeneic infected mice. Parasitemia was assessed at day 5, 7 and then every day by examination of Giemsa stained blood smears and was expressed as mean parasitemia. Body temperature was measured using an infrared thermometer (La Crosse Technology). Survival of mice was monitored and blood serum was collected when the temperature dropped down to 26°C. IL-1β was measured by ELISA with rat monoclonal and goat anti-mouse IL-1β. The detection limit was 6.25 pg/mL of IL-1β.

### Bone-marrow derived macrophages (BMDM)

Bone marrow cells were obtained by flushing the femurs and tibias from mice. Cells were used from fresh or from frozen marrows. Erythrocytes were lysed with 2 mL of NH_4_Cl (155 mM) in Tris/HCl (10 mM), pH 7.2 (9∶1 solution)/mouse. Bone marrow cells were adjusted to 7×10^6^ cells/10 mL and plated in 100 mm dishes with RPMI-1640 medium supplemented with 1% of PSG, 10% FBS and 30% (v/v) L929 cell culture supernatant. The supernatants of bone marrow cells were changed every two days in order to renew the cytokines and nutrients. After 7 days, the culture dishes were washed with PBS and replaced by ice cold PBS, incubated on ice for 15 min and cells were vigorously detached. BMDM were adjusted to 1.5×10^6^/2 mL or 0.2×10^6^ cells/0.5 mL in RPMI medium supplemented with 5% FBS (Gibco) and 1% of PSG and plated in 6 well plates (IL-1β) or 24 wells plate (confocal). The next day, cells were washed with warm PBS (37°C) and replaced by 500 µL of Alpha MEM medium without FBS. Cells were, as indicated in figure legends, stimulated with Hz, MSU or infected with *Salmonella typhimurium* as described by Franchi *et al.* [53].

### Supernatant and cell extract, immunoprecipitation (IP) and SDS-PAGE/immunoblotting analysis

Supernatant and cell extract analysis: After designated incubation time, supernatants were collected and protein was precipitated with trichloroacetic acid at 10% final concentration. Precipitates were then dissolved in Tris/HCl 0.1 mM pH 8.0 and Laemmli sample load buffer. Cell extracts were obtained by lysing cells with Igepal 1% (for signaling, in 1× PBS, 20% Glycerol, 1× inhibitor protease cocktail, 2 mM Na_3_VO_4_ and 1 mM NaF) or triton 1% (for caspase-1, in TNE buffer: 10 mM Tris/HCl pH 7.5, 150 mM NaCl, 5 mM EDTA and 1.5× inhibitor protease cocktail). Whole supernatant protein and equal amount of protein or cell lysate were subjected to SDS-PAGE and immunoblot analysis.

IP: Cells lysates were extracted with lysis buffer (1% CHAPs detergent in TNE buffer, 1× inhibitor cocktail, 2 mM Na_3_VO_4_ and 1 mM NaF). Cells lysates were pre-incubated for two hours at 4°C with protein A/G-coupled agarose beads and 1 µg of unspecific matched isotype control antibody (Ab). Equal amount of protein were immunoprecipitated with protein A/G-coupled agarose beads or True Blot anti-rabbit Ig and 2 µg of specific or unspecific matched isotype control Ab overnight. Beads were spun down 3 times with lysis buffer and proteins were denatured in Laemmli load buffer.

SDS-PAGE/Immunoblot: Samples from supernatants, cell extracts or IP were subjected to 10% (signaling) or 15% (IL-1β and caspase-1) acrylamide gel (all reagents from Laboratoire Mat. Inc., Montreal, Qc, Canada) or 4–12% NuPAGE® gel (for p10 caspase-1 and IP, Invitrogen). After transfer onto PVDF membranes, they were subjected to immunoblot analysis with the indicated Ab and matched secondary HRP-conjugated Ab. In some experiments, optical density was determined using AlphaDigiDoc 1000 v3.2 software (Alpha Innotech corporation).

### Confocal microscopy

OVA uptake: THP-1 cells (0.2×10^6^ cells/coverslip 12 mm from Fisher) were treated with 10 µg of DQ-OVA in the absence or presence of Hz (200 µg/mL) or Silica (400 µg/mL) for 30 min, washed and incubated up to three hours. Laser settings were adjusted on DQ-OVA fluorescence emission that is stronger than hemozoin or silica. Phagosome: BMDM were fixed, permeabilized using 0.1% Triton X-100, and non-specific surface Fcγ-receptor binding were blocked as described [54]. For immunofluorescence experiments, cells were labelled with the rat anti-LAMP-1 Ab and an anti-rat AlexaFluor 568. DRAQ5 was used to visualize DNA. Cathepsin B activity: THP-1 cells (0.2×10^6^ cells/coverslip 12 mm from Fisher) were pre-treated for 30 min with 5 µM of piceatannol and stimulated or not with Hz (200 µg/mL). A cathepsin B substract (Arg-Arg)_2_ linked with cresyl violet were given 30 min before the end of incubation time and cleaved substract generated a red fluorescence. All coverslips (THP-1/OVA or BMDM) were mounted on slides with Fluoromount-G. Detailed analysis of protein localization on the phagosome was performed by using an oil immersion Nikon Plan Apo 100 (N.A. 1.4) objective mounted on a Nikon Eclipse E800 microscope equipped with a Bio-Rad Radiance 2000 confocal imaging system (Bio-Rad Laboratories, Hercules, CA).

### In vivo neutrophil recruitment

WT, IL-1β-, NLRP3-, ASC-, caspase-1- and NLRC4-deficient mice were injected intraperitoneally with 800 µg of hemozoin in 1 ml of endotoxin-free PBS. Control groups were injected with 1 mL of PBS. After six hours, the mice were euthanized and the peritoneal cavity was washed with 10 mL of PBS. Cells recovered from the peritoneum were counted and the percentage of neutrophils was determined from an H&E stain (DiffQuick; Dade Behring, Inc.) of a cytospun sample.

### Statistical analysis

Unpaired Student's t-test was used when comparing two groups and ANOVA/Bonferroni test when comparing more than two groups. The differences were considered significant when p<0.05. Survival curves for infected and control mice were compared using the Mantel-Haenszel test. Statistical analysis was performed using Prism 5.00 software (GraphPad, San Diego, Calif.).

## Supporting Information

Figure S1 Field Emission Gun Scanning Electron Microscopy pictures of native and synthetic Hemozoin. Native Hemozoin from *Plasmodium falciparum* and chemically synthesized Hemozoin. FEG-SEM pictures were acquired using a Hitachi S-4700 FEG-SEM. The samples were coated with Au/Pd of about 4 Å thickness prior to visualization at 2 kV and 10 mA. Bars scale = 1 µm.
(1.77 MB TIF)

Figure S2 Hz is not contaminated with DNA or RNA. Hemozoin (Hz - 200 µg), DNA or RNA controls were treated or not with Dnase or Rnase. After enzymes inactivation and extensive washes in PBS, Hz samples were submitted to agarose gel (A) or used to stimulate PMA-differentiated THP-1 cells (B). After different time of incubation, supernatant (SN) and cell extracts were collected and subjected to Western blot analysis with the indicated antibodies (B). Data show one experiment representative of two independent experiments.
(0.04 MB PDF)

Figure S3 Hz, but not *Salmonella typhimurium*, stimulated IL-1β in macrophages from NLRC4-deficient mice. BMDM from WT or NLRC4-deficient mice (1.5×10^6^ cells/mL) were stimulated or not with Hz (200 µg/mL) or infected *Salmonella typhimurium* (ST - 1/10). After 6 h (Hz) or 2 h (Salmonella) of incubation supernatant (SN) and cell extract were collected and subjected to Western blot analysis with the indicated antibodies. Data show one experiment representative of three independent experiments.
(0.09 MB PDF)

Figure S4 Syk phosphorylation is not induced by MSU and LPS and is MyD88 independent. PMA-differentiated THP-1 cells (0.75×10^6^ cells/mL) were stimulated or not with MSU (100 µg/mL) or Hz (200 µg/mL) (A); BMDM (0.5×10^6^ cells/0.5 mL) were pre-treated or not with LPS (100 ng/mL) and stimulated or not with Hz (200 µg/mL) or LPS (100 ng/mL) (B). WT or MyD88-deficient macrophages (0.5×10^6^ cells/0.5 mL) were stimulated with Hz (200 µg/mL) or LPS (100 ng/mL) (C and D). After 10 (D) or 30 min (C) or indicated time of incubation cell extracts were collected and subjected to Western blot analysis with the indicated antibodies. Data show one experiment representative of at least three independent experiments.
(0.07 MB PDF)

Figure S5 IL-1β production and Syk phosphorylation induced by Hemozoin depends on intact lipid rafts. PMA-differentiated THP-1 cells (0.75×10^6^ cells/0.5 mL) were pre-treated with lipid raft disruptor MβCD and stimulated with Hz (200 µg/mL) for six hours (A) or 30 minutes (B). Supernatant (SN) and cell extracts were subjected to Western blot analysis with the indicated antibodies. Data show one experiment representative of three independent experiments. (C) PMA-differentiated THP-1 cells (0.2×10^6^ cells/0.5 mL) were pre-treated or not with 2 µM of MβCD and incubated in the presence or absence of 200 µg/mL of Hz (green) for 5 minutes. Cells were stained with cholera toxin B (red). Data shown are images obtained by confocal microscopy from one representative experiment of two independent experiments. Arrow shows Hz and lipid raft co-localization. Scale bars = 5 µm.
(0.07 MB PDF)
