# Correction: Malarial Hemozoin Activates the NLRP3 Inflammasome through Lyn and Syk Kinases

**DOI:** 10.1371/annotation/abca067d-b82b-4de6-93c5-0fcc38e3df05

**Published:** 2009-09-21

**Authors:** Marina Tiemi Shio, Stephanie C. Eisenbarth, Myriam Savaria, Adrien F. Vinet, Marie-Josée Bellemare, Kenneth W. Harder, Fayyaz S. Sutterwala, D. Scott Bohle, Albert Descoteaux, Richard A. Flavell, Martin Olivier

The first author's name in the article citation was incorrectly written. The correct name is "Shio MT."

