# Supplementary figures and images for: Malarial Hemozoin Activates the NLRP3 Inflammasome through Lyn and Syk Kinases
Source: PLoS Pathog. 2009 Aug 21;5(8):e1000559. doi: 10.1371/journal.ppat.1000559 (PMC2722371; doi:10.1371/journal.ppat.1000559)

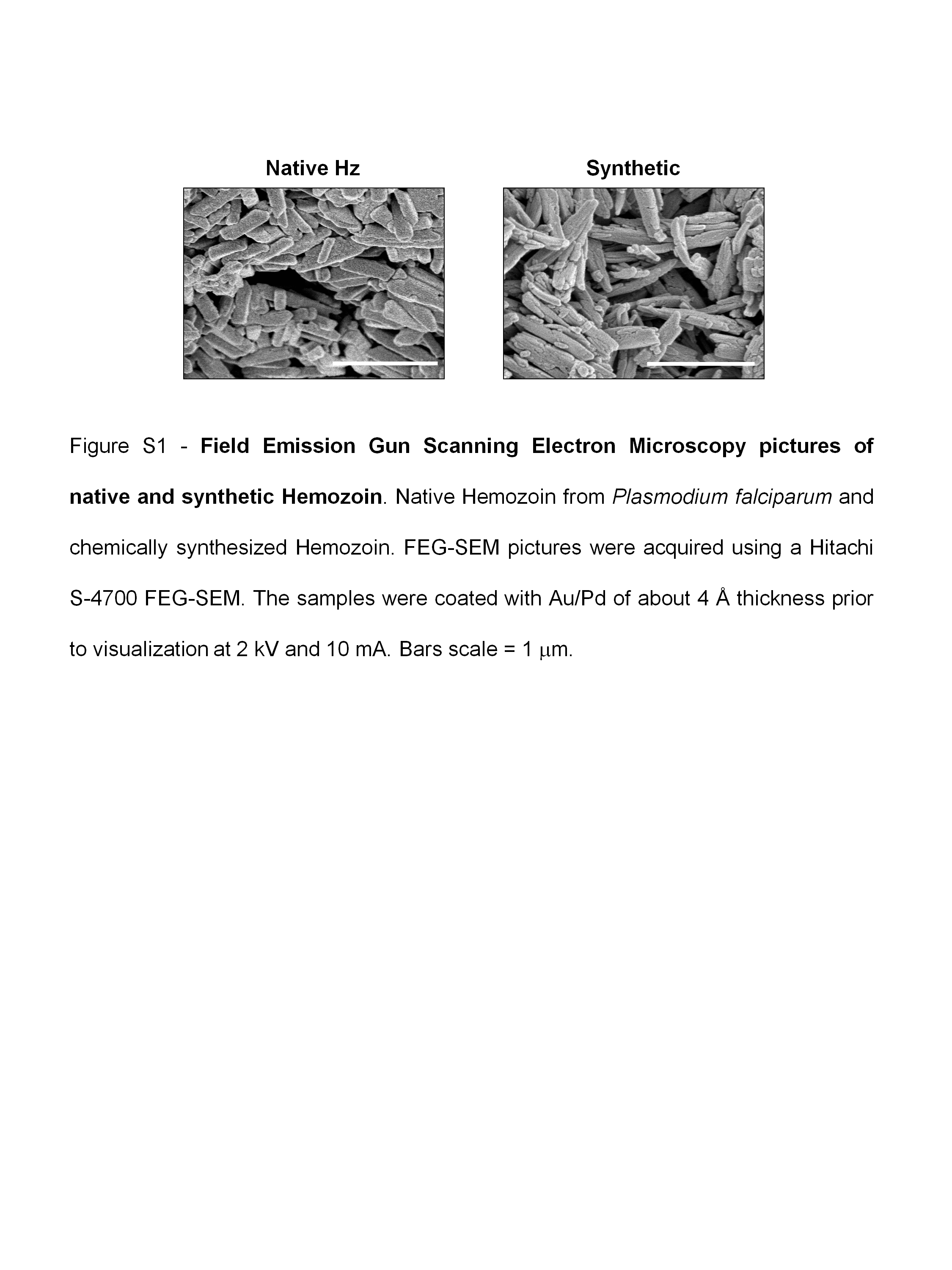

Supplement: Figure S1 — Field Emission Gun Scanning Electron Microscopy pictures of native and synthetic Hemozoin. Native Hemozoin from Plasmodium falciparum and chemically synthesized Hemozoin. FEG-SEM pictures were acquired using a Hitachi S-4700 FEG-SEM. The samples were coated with Au/Pd of about 4 Å thickness prior to visualization at 2 kV and 10 mA. Bars scale = 1 µm. (1.77 MB TIF) [file ppat.1000559.s001.tif]
