# Supplementary material for: Malarial Hemozoin Activates the NLRP3 Inflammasome through Lyn and Syk Kinases
Source: PLoS Pathog. 2009 Aug 21;5(8):e1000559. doi: 10.1371/journal.ppat.1000559 (PMC2722371; doi:10.1371/journal.ppat.1000559)
Supplement: Figure S2 — Hz is not contaminated with DNA or RNA. Hemozoin (Hz - 200 µg), DNA or RNA controls were treated or not with Dnase or Rnase. After enzymes inactivation and extensive washes in PBS, Hz samples were submitted to agarose gel (A) or used to stimulate PMA-differentiated THP-1 cells (B). After different time of incubation, supernatant (SN) and cell extracts were collected and subjected to Western blot analysis with the indicated antibodies (B). Data show one experiment representative of two independent experiments. (0.04 MB PDF) [file ppat.1000559.s002.pdf]

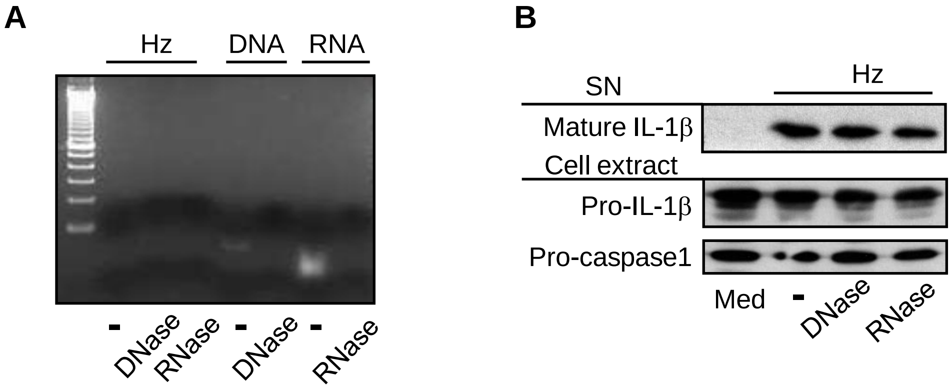

Figure S2 - **Hz is not contaminated with DNA or RNA.** Hemozoin (Hz - 200 µg), DNA or RNA controls were treated or not with Dnase or Rnase. After enzymes inactivation and extensive washes in PBS, Hz samples were submitted to agarose gel (**A**) or used to stimulate PMA-differentiated THP-1 cells (**B**). After different time of incubation, supernatant (SN) and cell extracts were collected and subjected to Western blot analysis with the indicated antibodies (**B**). Data show one experiment representative of two independent experiments.
