# Supplementary material for: Malarial Hemozoin Activates the NLRP3 Inflammasome through Lyn and Syk Kinases
Source: PLoS Pathog. 2009 Aug 21;5(8):e1000559. doi: 10.1371/journal.ppat.1000559 (PMC2722371; doi:10.1371/journal.ppat.1000559)
Supplement: Figure S3 — Hz, but not Salmonella typhimurium, stimulated IL-1β in macrophages from NLRC4-deficient mice. BMDM from WT or NLRC4-deficient mice (1.5×10^6^ cells/mL) were stimulated or not with Hz (200 µg/mL) or infected Salmonella typhimurium (ST - 1/10). After 6 h (Hz) or 2 h (Salmonella) of incubation supernatant (SN) and cell extract were collected and subjected to Western blot analysis with the indicated antibodies. Data show one experiment representative of three independent experiments. (0.09 MB PDF) [file ppat.1000559.s003.pdf]

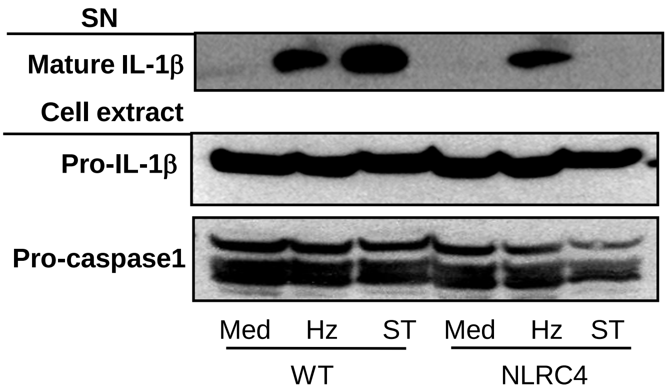

Figure S3 - **Hz, but not *Salmonella typhimurium*, stimulated IL-1β in macrophages from NLRC4-deficient mice**. BMDM from WT or NLRC4-deficient mice ($1.5x10^6$ cells/mL) were stimulated or not with Hz (200 μg/mL) or infected *Salmonella typhimurium* (ST - 1/10). After 6 h (Hz) or 2 h (Salmonella) of incubation supernatant (SN) and cell extract were collected and subjected to Western blot analysis with the indicated antibodies. Data show one experiment representative of three independent experiments.
