# Supplementary material for: Malarial Hemozoin Activates the NLRP3 Inflammasome through Lyn and Syk Kinases
Source: PLoS Pathog. 2009 Aug 21;5(8):e1000559. doi: 10.1371/journal.ppat.1000559 (PMC2722371; doi:10.1371/journal.ppat.1000559)
Supplement: Figure S4 — Syk phosphorylation is not induced by MSU and LPS and is MyD88 independent. PMA-differentiated THP-1 cells (0.75×10^6^ cells/mL) were stimulated or not with MSU (100 µg/mL) or Hz (200 µg/mL) (A); BMDM (0.5×10^6^ cells/0.5 mL) were pre-treated or not with LPS (100 ng/mL) and stimulated or not with Hz (200 µg/mL) or LPS (100 ng/mL) (B). WT or MyD88-deficient macrophages (0.5×10^6^ cells/0.5 mL) were stimulated with Hz (200 µg/mL) or LPS (100 ng/mL) (C and D). After 10 (D) or 30 min (C) or indicated time of incubation cell extracts were collected and subjected to Western blot analysis with the indicated antibodies. Data show one experiment representative of at least three independent experiments. (0.07 MB PDF) [file ppat.1000559.s004.pdf]

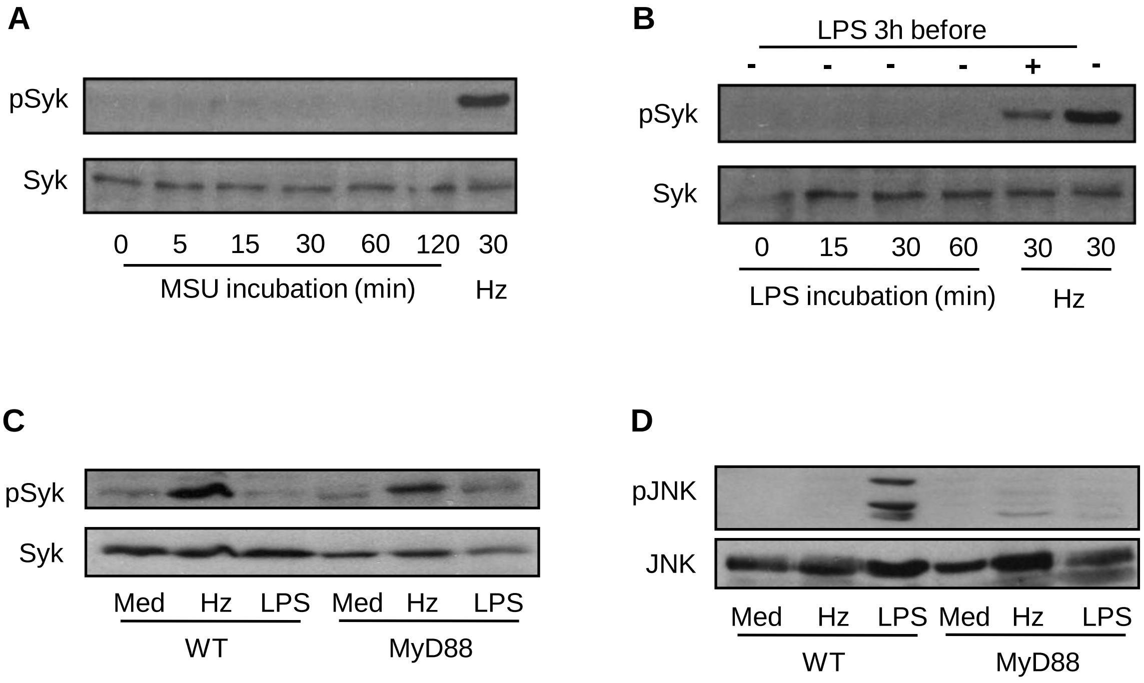

Figure S4 - **Syk phosphorylation is not induced by MSU and LPS and is MyD88 independent**. PMA-differentiated THP-1 cells (0.75x10^6 cells/mL) were stimulated or not with MSU (100 μg/mL) or Hz (200 μg/mL) (**A**); BMDM (0.5x10^6 cells/0.5 mL) were pre-treated or not with LPS (100ng/mL) and stimulated or not with Hz (200 μg/mL) or LPS (100 ng/mL) (**B**). WT or MyD88-deficient macrophages (0.5x10^6 cells/0.5 mL) were stimulated with Hz (200 μg/mL) or LPS (100 ng/mL) (**C** and **D**). After 10 (**D**) or 30 min (**C**) or indicated time of incubation cell extracts were collected and subjected to Western blot analysis with the indicated antibodies. Data show one experiment representative of at least three independent experiments.
