# Supplementary material for: Malarial Hemozoin Activates the NLRP3 Inflammasome through Lyn and Syk Kinases
Source: PLoS Pathog. 2009 Aug 21;5(8):e1000559. doi: 10.1371/journal.ppat.1000559 (PMC2722371; doi:10.1371/journal.ppat.1000559)
Supplement: Figure S5 — IL-1β production and Syk phosphorylation induced by Hemozoin depends on intact lipid rafts. PMA-differentiated THP-1 cells (0.75×10^6^ cells/0.5 mL) were pre-treated with lipid raft disruptor MβCD and stimulated with Hz (200 µg/mL) for six hours (A) or 30 minutes (B). Supernatant (SN) and cell extracts were subjected to Western blot analysis with the indicated antibodies. Data show one experiment representative of three independent experiments. (C) PMA-differentiated THP-1 cells (0.2×10^6^ cells/0.5 mL) were pre-treated or not with 2 µM of MβCD and incubated in the presence or absence of 200 µg/mL of Hz (green) for 5 minutes. Cells were stained with cholera toxin B (red). Data shown are images obtained by confocal microscopy from one representative experiment of two independent experiments. Arrow shows Hz and lipid raft co-localization. Scale bars = 5 µm. (0.07 MB PDF) [file ppat.1000559.s005.pdf]

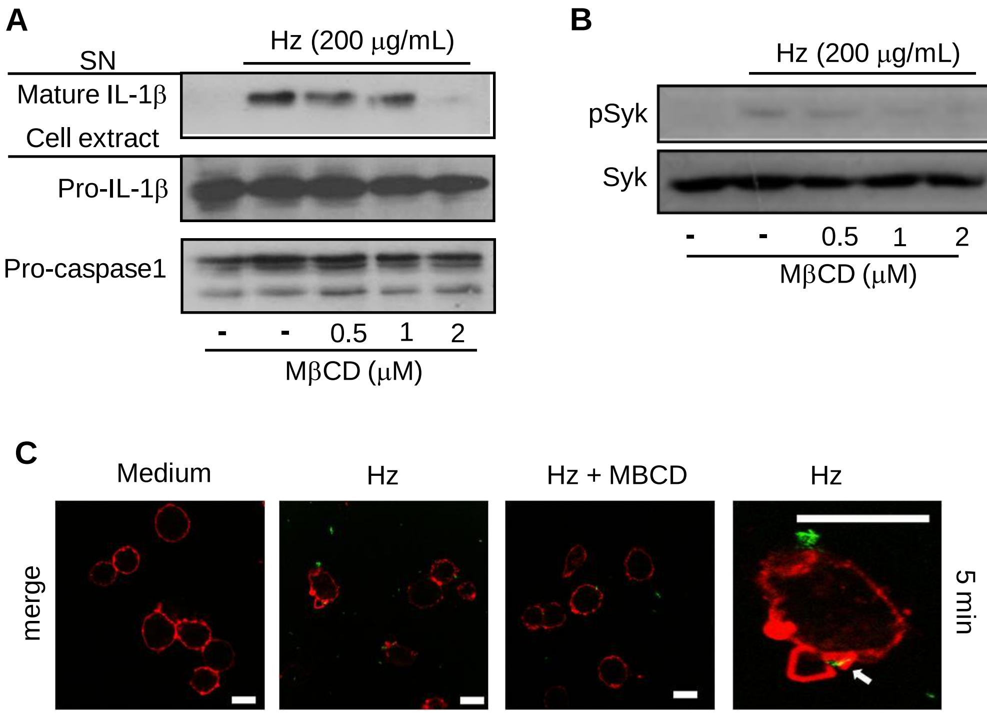

Figure S5 - **IL-1β production and Syk phosphorylation induced by Hemozoin depends on intact lipid rafts**. PMA-differentiated THP-1 cells ($0.75x10^6$ cells/0.5 mL) were pre-treated with lipid raft disruptor MβCD and stimulated with Hz (200 μg/mL) for six hours (**A**) or 30 minutes (**B**). Supernatant (SN) and cell extracts were subjected to Western blot analysis with the indicated antibodies. Data show one experiment representative of three independent experiments. (**C**) PMA-differentiated THP-1 cells ($0.2x10^6$ cells/0.5 mL) were pre-treated or not with 2 μM of MbCD and incubated in the presence or absence of 200 μg/mL of Hz (green) for 5 minutes. Cells were stained with cholera toxin B (red). Data shown are images obtained by confocal microscopy from one representative experiment of two independent experiments. Arrow shows Hz and lipid raft co-localization. Scale bars = 5 μm
